# Social Volunteering in Aging Adults Increases Regions of the Amygdala and Correlates With Enhanced Generativity

**DOI:** 10.1093/geroni/igaa057.2881

**Published:** 2020-12-16

**Authors:** Michelle Carlson

**Affiliations:** Johns Hopkins Bloomberg School of Public Health, Baltimore, Maryland, United States

## Abstract

The Brain Health Study (BHS) of the Baltimore Experience Corps Trial (BECT) examined whether a randomized, controlled trial of an intergenerational social volunteer program, entitled Experience Corps, increased subregions of the amygdala related to socioemotional memory and risk for Alzheimer’s disease in aging adults. We further assessed functional correlates of these intervention-related changes and changes in aging adults’ developmental need to be generative, or, to give back to the well-being of others. The BHS simultaneously randomized 112 men and women (59 intervention; 53 control) within BECT to evaluate intervention impact on biomarkers of brain health at baseline and annual follow-ups during the two-year trial. Intention-to-treat analyses revealed program-specific increases in the shape of the centromedial and basomedial regions of the left amygdala (p’s≤0.05 adjusted), which were correlated with increases in generativity (p’s =0.06). Meaningful social engagement buffered amygdalar declines important to preservation of emotionally salient memory and risk for dementia. Part of a symposium sponsored by Brain Interest Group.

